# Supplementary material for: A 3D-CT Analysis of Femoral Symmetry—Surgical Implications
Source: J Clin Med. 2020 Nov 3;9(11):3546. doi: 10.3390/jcm9113546 (PMC7693666; doi:10.3390/jcm9113546)
Supplement: Supplementary file 1 [file jcm-09-03546-s001.pptx]

## Slide 1
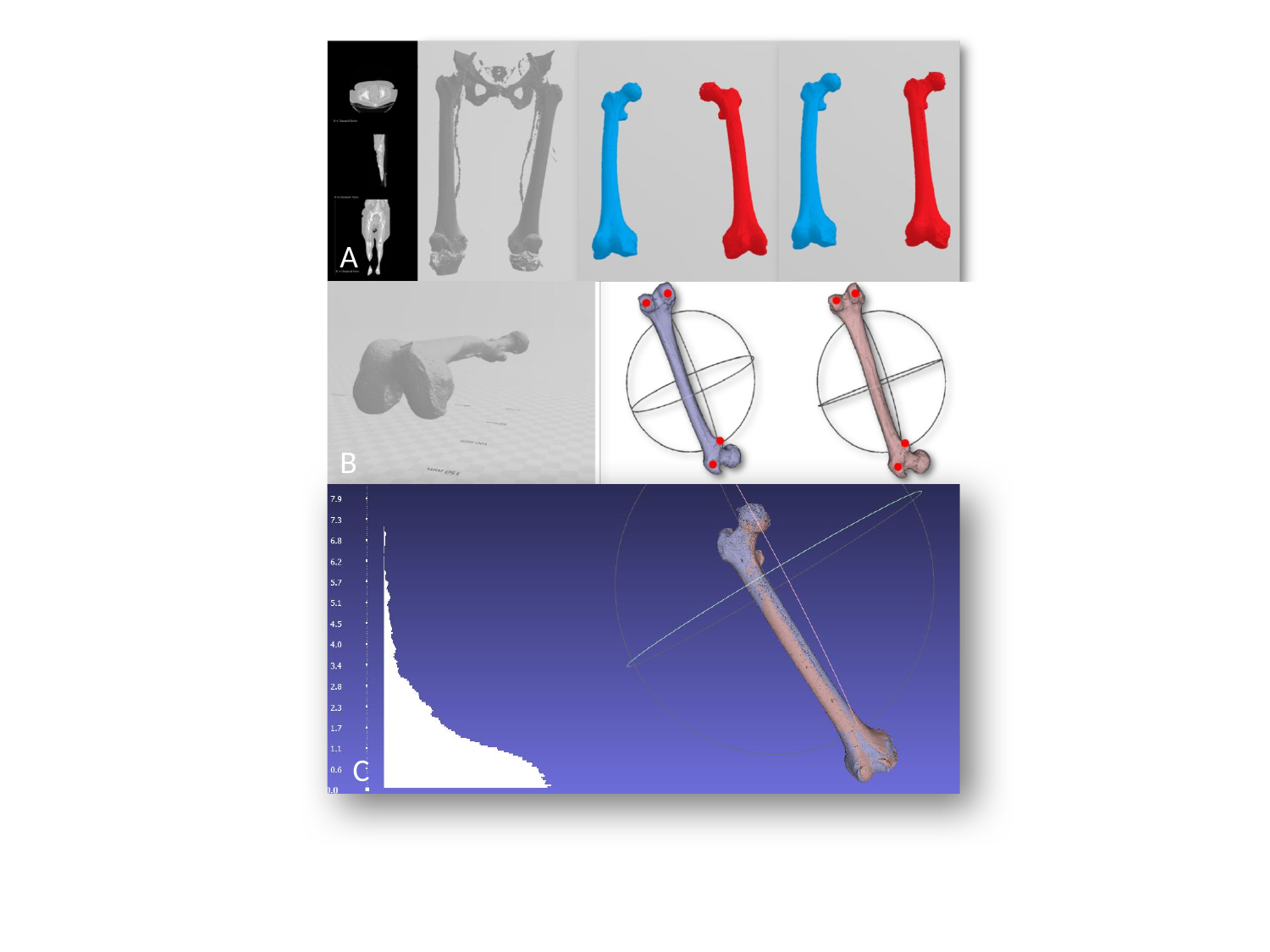

A
B
C

## Slide 2
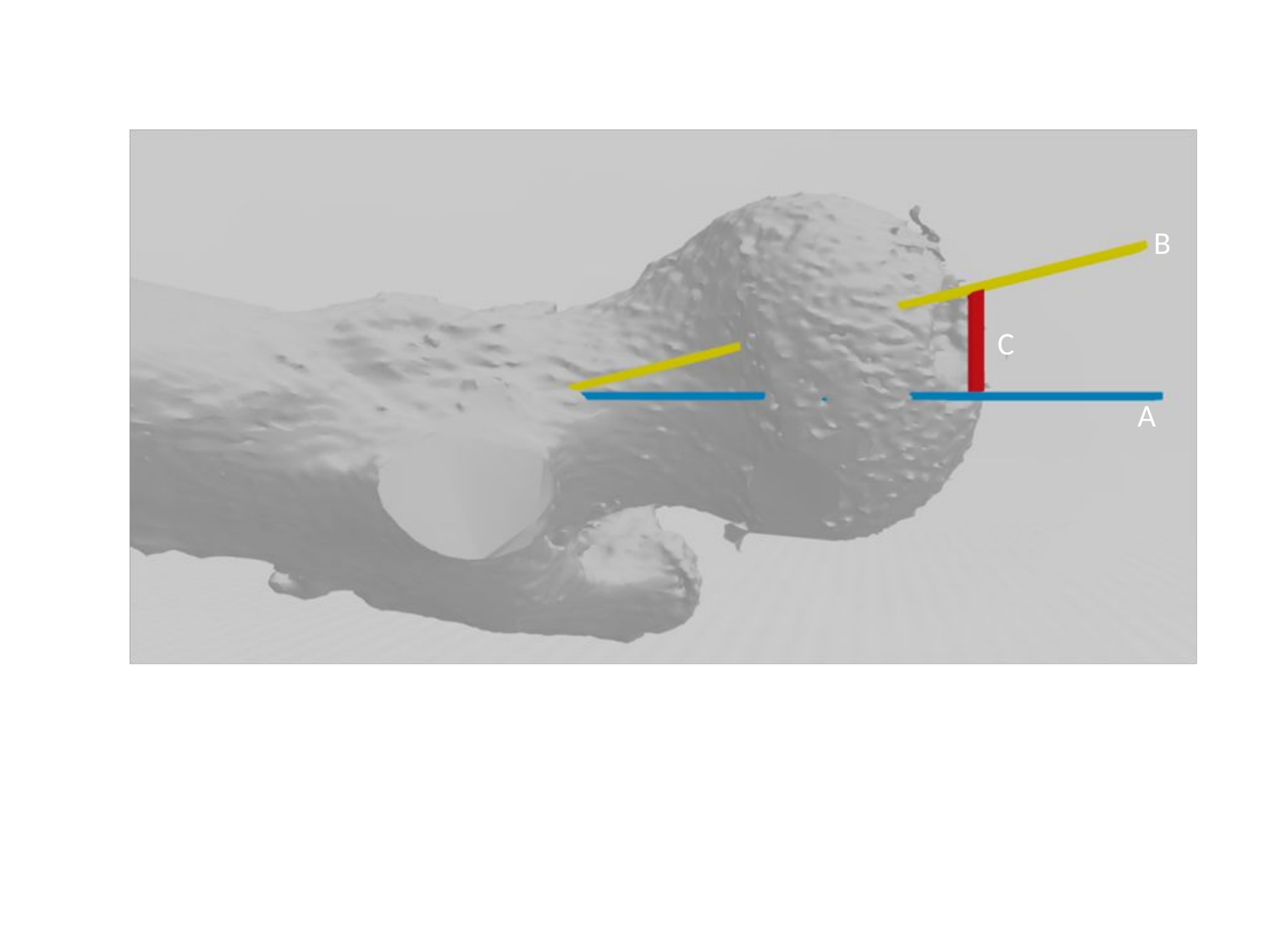

B
C
A

## Slide 3
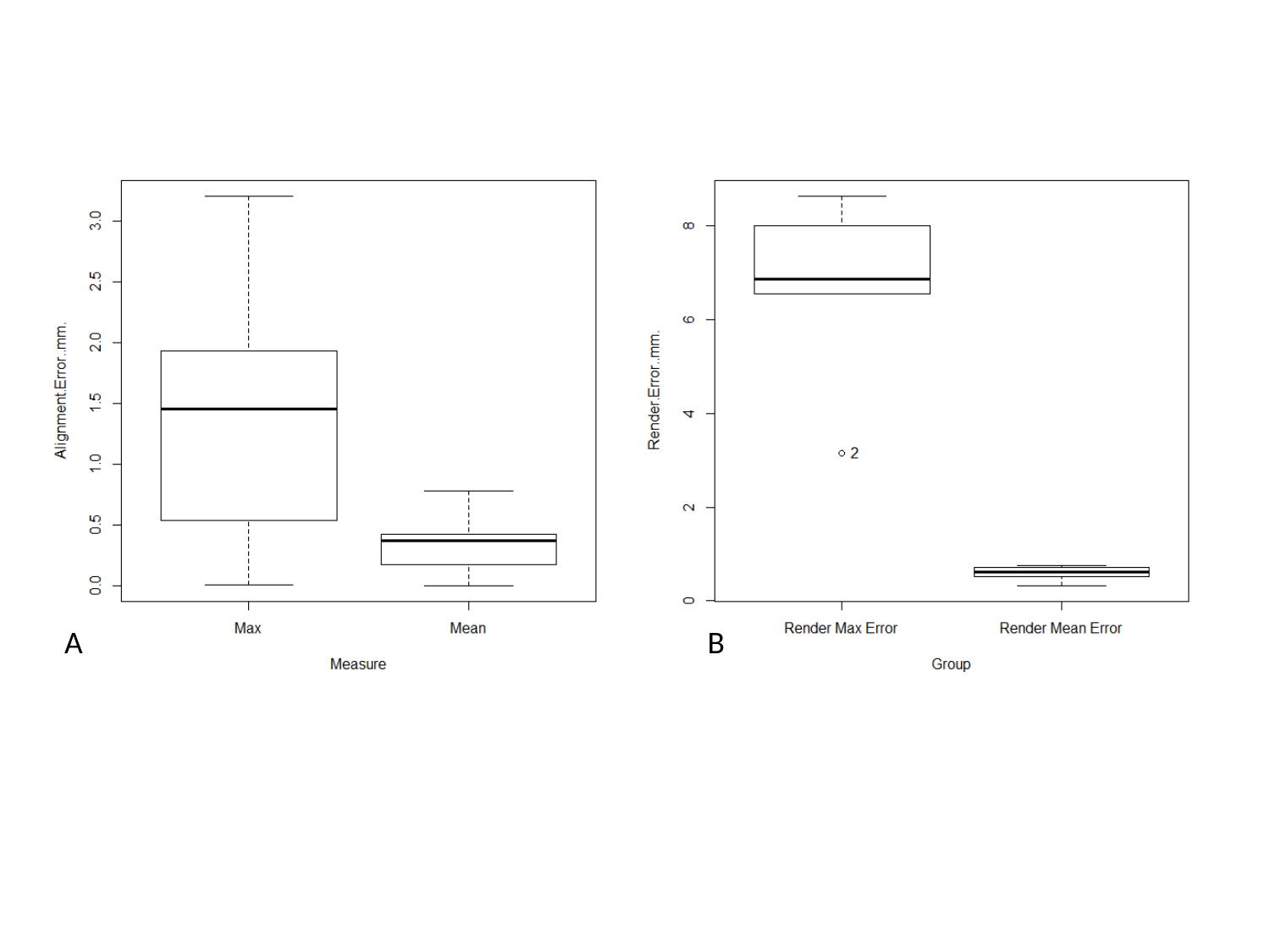

A
B

## Slide 4
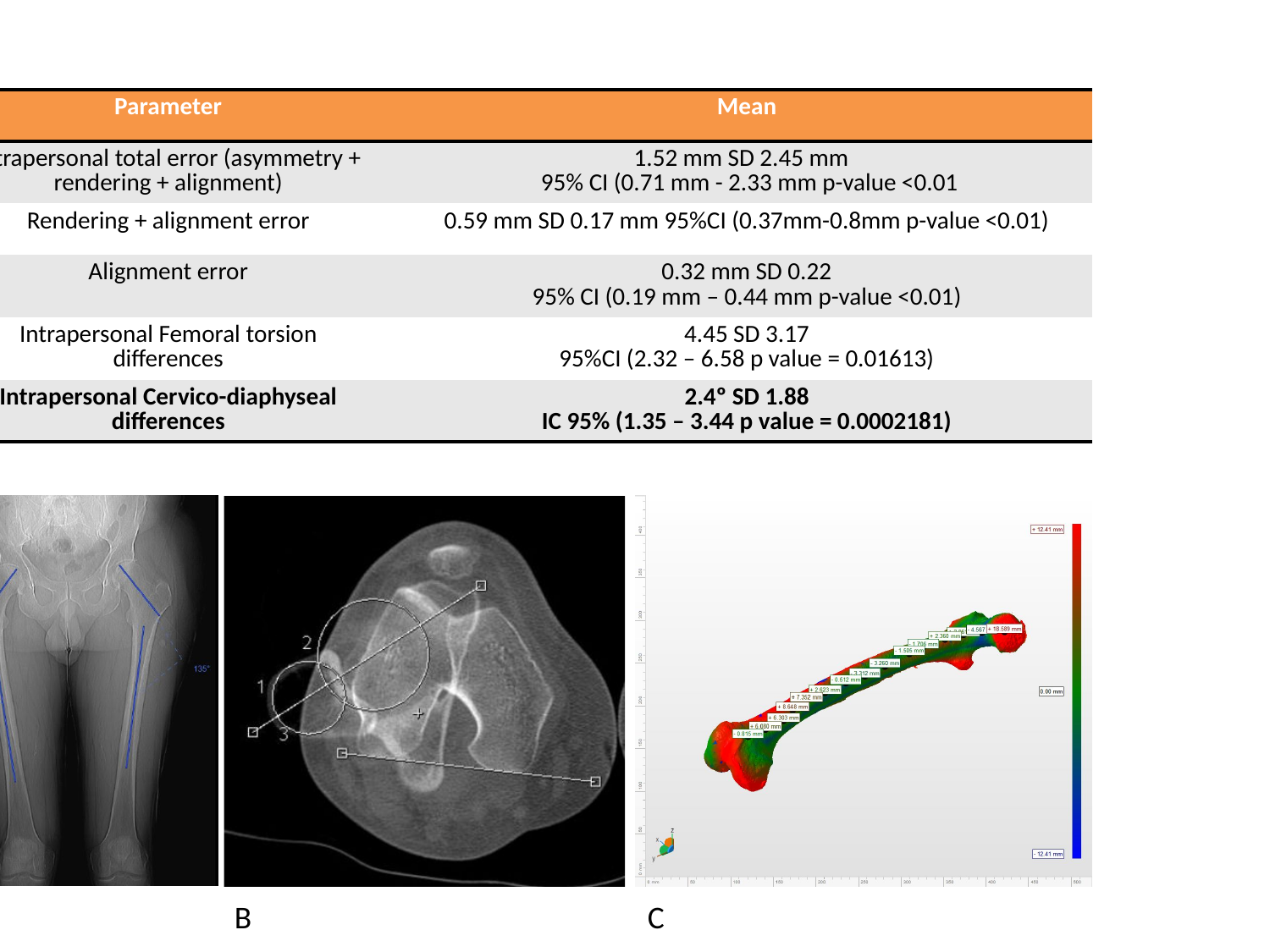

| Parameter | Mean |
| --- | --- |
| Intrapersonal total error (asymmetry + rendering + alignment) | 1.52 mm SD 2.45 mm 95% CI (0.71 mm - 2.33 mm p-value ˂0.01 |
| Rendering + alignment error | 0.59 mm SD 0.17 mm 95%CI (0.37mm-0.8mm p-value ˂0.01) |
| Alignment error | 0.32 mm SD 0.22 95% CI (0.19 mm – 0.44 mm p-value ˂0.01) |
| Intrapersonal Femoral torsion differences | 4.45 SD 3.17 95%CI (2.32 – 6.58 p value = 0.01613) |
| Intrapersonal Cervico-diaphyseal differences | 2.4º SD 1.88 IC 95% (1.35 – 3.44 p value = 0.0002181) |
A
B
C

## Slide 5
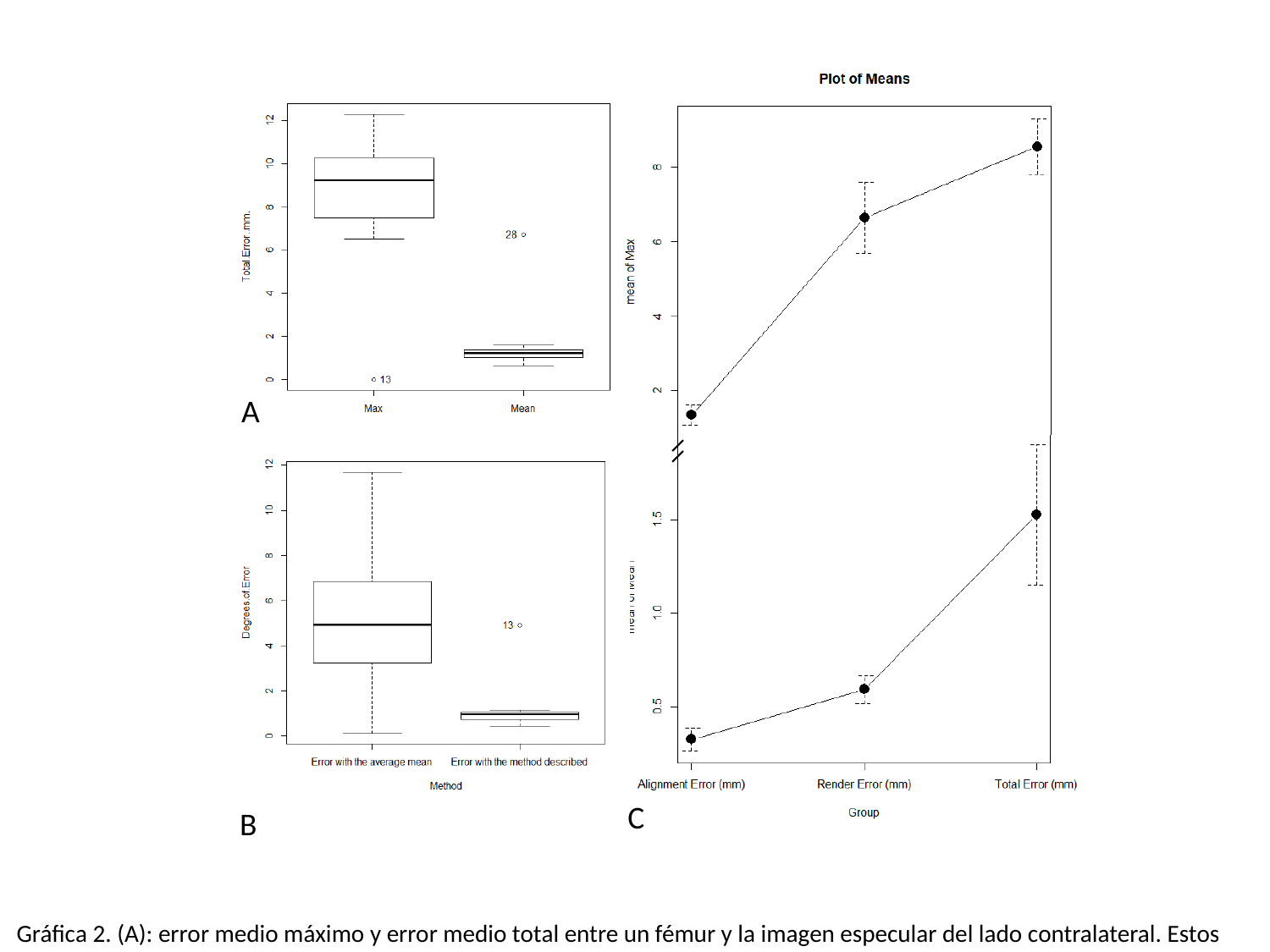

A
C
B
Gráfica 2. (A): error medio máximo y error medio total entre un fémur y la imagen especular del lado contralateral. Estos errores incluyen el error por asimetría, el error por renderizado y el error por alineación, intrínsecos al procesamiento de los biomodelos. (B): variabilidad interpersonal (representada como la media de las diferencias de versión femoral de los pacientes respecto la media de la serie) y variabilidad intrapersonal (representada como la media de las diferencias de versión femoral de los pacientes respecto a su lado contralateral con el método descrito). (C): representación de la contribución al error total del error por alineación y del error por renderizado.

## Slide 6
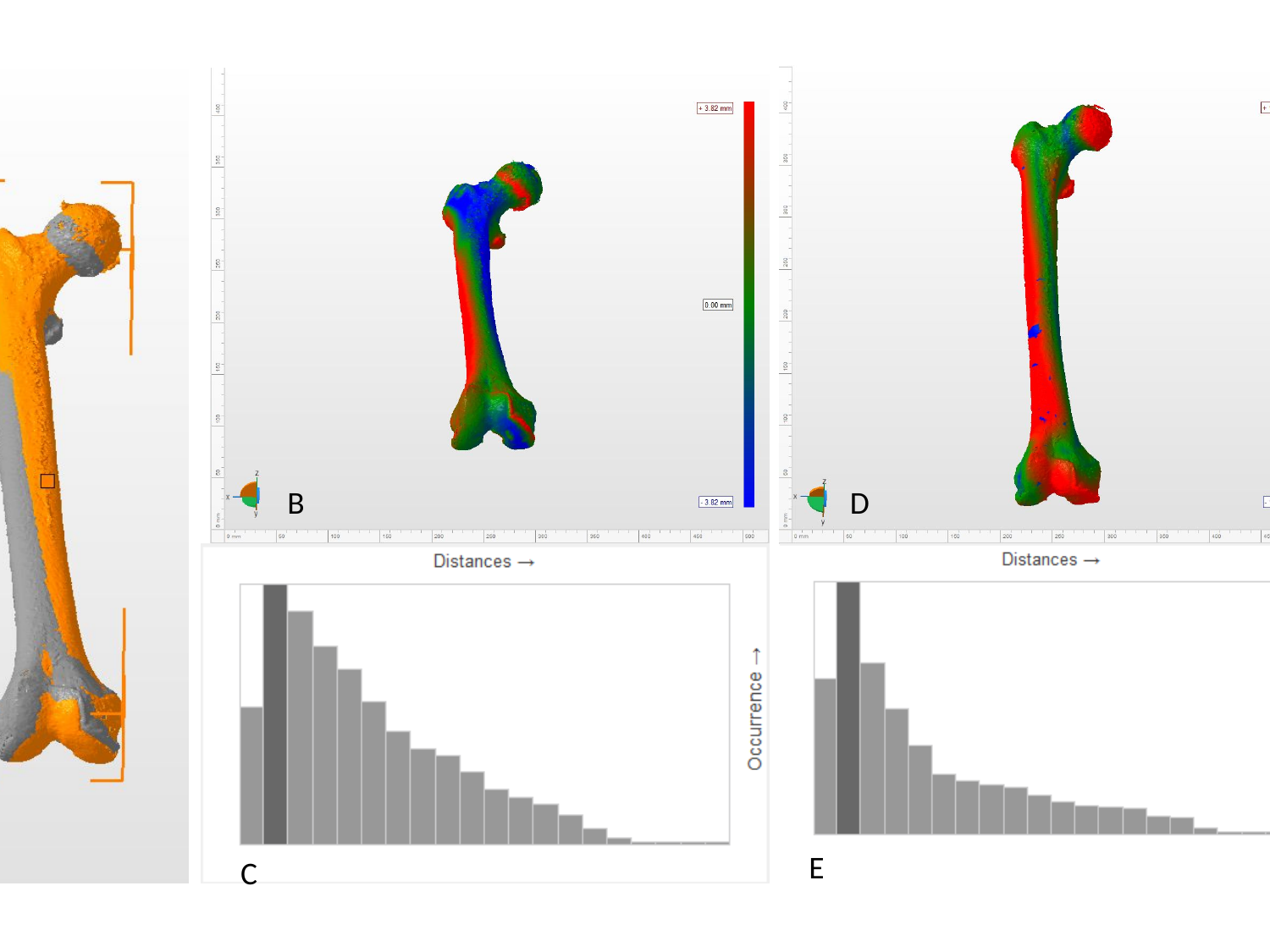

B
D
A
E
C
